# Colony adaptive response to simulated heat waves and consequences at the individual level in honeybees (*Apis mellifera*)

**DOI:** 10.1038/s41598-017-03944-x

**Published:** 2017-06-19

**Authors:** Célia Bordier, Hélène Dechatre, Séverine Suchail, Mathilde Peruzzi, Samuel Soubeyrand, Maryline Pioz, Michel Pélissier, Didier Crauser, Yves Le Conte, Cédric Alaux

**Affiliations:** 1 Abeilles et Environnement, INRA, 84914 Avignon, France; 2 Institut Méditerranéen de Biodiversité et d’Ecologie marine et continentale, Pôle Agrosciences, 301 rue Baruch de Spinoza, 84916 Avignon, France; 3 BioSP, INRA, 84914 Avignon, France

## Abstract

Since climate change is expected to bring more severe and frequent extreme weather events such as heat waves, assessing the physiological and behavioural sensitivity of organisms to temperature becomes a priority. We therefore investigated the responses of honeybees, an important insect pollinator, to simulated heat waves (SHW). Honeybees are known to maintain strict brood thermoregulation, but the consequences at the colony and individual levels remain poorly understood. For the first time, we quantified and modelled colony real-time activity and found a 70% increase in foraging activity with SHW, which was likely due to the recruitment of previously inactive bees. Pollen and nectar foraging was not impacted, but an increase in water foragers was observed at the expense of empty bees. Contrary to individual energetic resources, vitellogenin levels increased with SHW, probably to protect bees against oxidative stress. Finally, though immune functions were not altered, we observed a significant decrease in deformed wing virus loads with SHW. In conclusion, we demonstrated that honeybees could remarkably adapt to heat waves without a cost at the individual level and on resource flow. However, the recruitment of backup foraging forces might be costly by lowering the colony buffering capacity against additional environmental pressures.

## Introduction

Climate change is characterized by an increase in the world mean surface temperature^1^ and has altered the phenology, geographic distribution and population abundance in many species during the past half-century^2^. However, these phenological and distribution shifts can vary greatly in direction and magnitude depending on the species^3, 4^, making it difficult to predict the response of species. Therefore, to better understand the impact of climate change on species, one approach is to integrate the study of biological traits with environmental variation and quantify the physiological and behavioural sensitivity of the organisms to temperature^5^.

Defining the thermal sensitivity of organisms with a limited ability to regulate their internal body temperature, such as many insects and other ectotherms, is especially important since they are the most likely to respond to climate change. In addition, if outside of tropical areas, they can tolerate winter conditions by entering lethargic stages (e.g., diapause), they cannot readily escape from stressful hot summers^5^. Therefore, they might be susceptible to the increase in climate variability related to climate change, such as heat waves^6^, which is when the daily maximum temperature exceeds for more than five consecutive days the maximum normal temperature by 5 °C (the normal period being 1961–1990 period)^6^. These heat waves have been reported to severely affect some insect species (increased mortality) such as flies, butterflies and bees^7–10^, which are vital to supporting natural biodiversity and agroecosystems via pollination services^11^. Honeybees, also participating in this ecosystem service, may be affected by heat waves, but due to their social buffering capacities, they are likely less susceptible and more resilient to environmental pressures than other insects^12^. However, how they adapt and the cost of that adaption is far from being understood.

In honeybee colonies, brood temperature is strictly controlled within a temperature range of 33 to 36 °C^13^, and temperature control is even more precise during the pupal period (35 ± 0.5 °C)^14, 15^. Indeed, if adult bees are rather eurytherm, the brood is stenotherm^13^. Maintaining this optimal temperature window is crucial for the colony since extended deviations are known to increase mortality^16^, cause deformities^17^ and affect the synaptic organization in the brain of adults bees^18^. In addition, pupal developmental temperature can also influence behavioural performances^19, 20^ and specialization^21^. Though the mechanisms by which environmentally induced changes in temperature are compensated within the colony are well known (endothermic heat production and evaporation of water by wing fanning to warm and cool down the brood, respectively^22, 23^), the consequences or costs at the colony and individual levels have been poorly investigated (e.g., alteration of resource foraging, task allocation, energetic resources). Investigating the response to heat waves is notably important since such extreme whether events are expected to increase in frequency and magnitude in the future^24^.

We therefore exposed colonies to simulated heat waves (SHW) (2 °C above the optimal temperature of pupal development) and analysed their impact on foraging activity. An increase in water foraging was excepted to ensure evaporative cooling of the brood^25–27^, but we assessed whether it occurs at the expense of pollen and nectar foraging therefore decreasing resource income or if it simply involves an increase in global foraging activity without affecting resource income. Next, we determined the impact of simulated heat waves on adult energetic resources (circulating sugar levels and glycogen stores) since an increase in energy mobilization was expected in relation to a higher rate of evaporative cooling and foraging activity. Finally, it is known from previous studies in insects that changes in environmental temperature can affect immune functions^28, 29^ and can have major effects on host-parasite interactions by affecting the resistance of hosts to viruses^30, 31^, bacteria^32^, microsporidia^33^ and fungi^34, 35^. We therefore investigated whether SHW modified bee immune systems by quantifying the expression level of genes involved in humoural immunity (apidaecin 1, defensin 1) and cellular immunity (eater) or both (prophenoloxidase-PPO), as well as vitellogenin, which is also involved in bee immunity but has multifunctional roles (protection against oxidative stress, task specialization^36^). The levels of Deformed Wing Virus (DWV), a highly prevalent and pathogenic virus in honeybees^37^, were also determined with bee immune function and following SHW treatments.

## Methods

### Experimental setup and heat wave simulation

Experiments were performed during the springs of 2015 and 2016 at the Institut National de la Recherche Agronomique (INRA) in Avignon (France) with hybrid honeybee colonies (a mix of *Apis mellifera ligustica* and *Apis mellifera mellifera*). Each year, three colonies of similar size and strength were set in nuclei hives composed of 5 Dadant frames. Hives were placed in an indoor apiary (14 m^2^) to control their environmental temperature, but were connected to the outside via the entrance, so that bees were free to fly. Each colony originated from a full sized colony that was split in half, and was composed of 3 brood frames and 2 storage frames. The presence of eggs was regularly checked to make sure that the queen was actively laying eggs. During the experiment, colonies did not outgrow their nuclei hives.

The indoor apiary was set at a constant temperature of 23 °C for 5 days, followed by 5 days of a daily simulated heat wave (SHW) at 37 °C; this temperature is 2 °C above the optimal temperature of pupal development and is typically recorded during the summer in the South-East of France (with average minimal temperature of 17 °C and regular peaks above 40 °C)^38^. This temperature jump might represent an extreme scenario, but our goal was to assess whether responding to relatively high temperature induces a cost to the colony (via the need for thermoregulation). To be as realistic as possible with the SHW, the temperature increase was initiated in the morning at 8 am at room temperature, reaching 37 °C around noon, and lasting until 8 pm. The overnight temperature was set back to 23 °C. We therefore obtained a ‘control’ temperature treatment of 5 days (SHW−) and 5 days of SHW (with a daily peak at 37 °C; SHW+). This 10-day temperature cycle was repeated 3 times (3 trials).

Temperatures inside each colony were recorded with thermo-tracers (Oceasoft, ±0.5 °C) placed on a central and storage frame. The temperature was recorded every 5 minutes and recovered using Thermo-Tracer version 3.1 (Oceasoft).

Colony activity and foraging behaviour (see below) were recorded every day, except for the first day of a new temperature regime for foraging behaviour. To assess the impact of SHW on bee physiology, bees were sampled on the last day of each temperature regime. In addition, we visually estimated the percentage of brood per frame side (capped and uncapped cells) at the end of each period of 5 days to obtain information on colony development. Brood percentages were then converted into number of cells considering a theoretical number of 4,000 brood cells per frame side (personal observation). The influence of SHW on bee physiology and colony activity was assessed in 2015. Variations in foraging activity and brood size were determined in 2016. Each year, all colonies were set up and evaluated at the same time.

### Impact of heat waves on colony activity

To assess the flight activity at the colony level, we equipped each colony with an optic bee counter developed in our laboratory (patent IDDN.FR.001.130013.000.R.P.2010.000.31235). This device, described in Dussaubat *et al*.^39^, allows for the continuous recording of the flight activity of all bees at the colony entrance by counting the number of out-going and in-coming bees. The cumulated activity was recorded every 5 minutes and automatically saved in an Excel file with a date and time. A colony of the same size as the indoor colonies was placed in the field next to the indoor apiary and was equipped with an optic bee counter. This colony was used, as an environmental control, to determine whether differences in colony activity could be attributed to variation in field conditions (e.g., climate, resources).

### Impact of heat waves on foraging behaviour

For each colony, the proportion of pollen foragers was determined by counting the number of bees coming back to the colony with or without pollen during a 5-minute period (total of 36,572 bees coming back to the colonies). Next, we sampled bees without pollen for 20 minutes to identify the type of resources they foraged (from 18 to 94 bees per colony and day). We applied dorso-ventral pressure on the abdomen of each sampled bee to make them regurgitate the crop content^40^. Bees that came back with an empty crop were defined as empty bees. When bees regurgitated, the liquid was collected with 10 or 20 µL microcapillary tubes (Hirschmann®, Ringcaps®). To determine the volume, the liquid height was measured in millimetres and converted into microliters. The concentration of sugar in the liquid was measured, as a percentage of saccharose equivalent, using manual refractometers (Bellingham & Stanley Ltd, Tunbridge Wells, UK: 0–50 Brix and 45–80 °Brix). As in Reetz *et al*.^41^, bees were identified as water and nectar foragers when the saccharose concentration of the crop content was below or above 15%, respectively.

The foraging behaviour of bees (pollen, nectar and water foragers) was assessed 3 times each afternoon on the 3 colonies (colonies were analysed serially each time and order was randomized each day), and we obtained a total of 5,719 sampled bees.

### Impact of heat waves on bee physiology

#### Energetic resources

The impact of heat waves on energetic resources was assessed by quantifying glycogen reserves and circulating carbohydrate levels (glucose, trehalose and fructose) in bees. Bees were sampled on both brood and storage frames (containing pollen and honey). Haemolymph sampling was performed immediately after bee collection. For that purpose, we pricked bees between the third and fourth tergite with a needle and collected 3 to 5 µL of haemolymph with microcapillary tubes (Ringcaps®). Whole bees and haemolymph samples were stored at −20 °C. We analysed 6 bees per colony and replicate, giving a final sample size of 54 bees per temperature condition and type of frame.

The glycogen level in the whole bee was quantified by spectrophotometry using the Glucose RTU^TM^ method (BioMérieux SA) as described in Bordier *et al*.^42^. Briefly, the absorbance was determined at 505 nm, and the amount of glucose was calculated from a standard curve obtained from serial dilutions of pure glucose treated within the same conditions.

The haemolymph sugar levels were quantified for each bee by high-pressure ion chromatography (HPIC) Dionex ICS-3000 following the protocol described by Rusch *et al*.^43^. The curves of sugars were obtained for trehalose, glucose, and fructose. For the post chromatography analysis, peaks areas were converted into sugar concentrations.

#### Immune gene expression and DWV loads

To have an overview at the colony level of physiological changes, bees were sampled on brood frames (containing bees from all successive age classes). Bees were immediately stored at −80 °C. We analysed 3 pools of 10 bees per colony and replicate, giving a final sample size of 27 pools per temperature condition. Analyses were performed on abdomens since antimicrobial peptides and vitellogenin are mainly synthetized in the abdominal fat body. Total RNA was extracted from the abdomens by homogenizing them in 900 µL of Qiazol reagent (Qiagen). To quantify the gene expression and DWV loads in each pool of bees, RNA extraction, cDNA synthesis and quantitative PCR were conducted using the protocol described in Di Pasquale *et al*.^44^. Cycle threshold values of vitellogenin, apidaecin 1, defensin 1, eater, and prophenoloxidase were normalized to the geometric mean of the housekeeping gene actin and eIF*3*-S8 using the comparative quantification method (delta Ct method) for genes. Those housekeeping genes were previously found to remain unchanged upon heat stress^42^. For DWV, external standards of known concentration were obtained from 10-fold serial dilutions and used for absolute quantification. Non-template controls were also included on each plate. Primer sequences are reported in Table S1.

### Statistical analysis

All statistics were performed using the statistical software R version 3.2.1^45^.

The activity of the colony was measured across time by the number of exits *N*_*t*_ between times *t*-1 and *t*, which are separated by a time lag of 5 minutes. The number of exits was modelled by the following autoregressive conditional Poisson model:

1 $${N}_{t} \sim {\rm{Poisson}}\,(\alpha +\beta {N}_{t-1}+{\gamma }_{1}({d}_{t},t)+{\gamma }_{2}({d}_{t},t)),$$

where α is a basic number of exits, β*N*_*t*−*1*_ is an auto-correlation term, γ_1_(*d*_*t*_, *t*) is an additional morning effect at day *d*_*t*_ (this effect applies only if *t* belongs to the period [8:00–12:00] of day *d*_*t*_) and γ_2_(*d*_*t*_, *t*) is an additional afternoon effect at day *d*_*t*_ (this effect applies only if *t* belongs to the period [12:00–20:00] of day *d*_*t*_). More precisely, the basic number of exits α is defined as the expected number of exits if *N*_*t*−*1*_ = 0 and *t* is outside the period [8:00–20:00], α + γ_1_(*d*_*t*_, *t*) is the expected number of exits if *N*_*t*−*1*_ = 0 and *t* belongs to the period [8:00–12:00], and α + γ_2_ (*d*_*t*_, *t*) is the expected number of exits if *N*_*t*−*1*_ = 0 and *t* belongs to the period [12:00–20:00]. However, since *N*_*t*−*1*_ is not 0 in general, the expected number of exits is the sum of (i) either α, α + γ_1_ (*d*_*t*_, *t*) or α + γ_2_(*d*_*t*_, *t*), depending on the period during the day and (ii) the auto-correlation term β*N*_*t*−*1*_. In the model, the term β*N*_*t*−*1*_ reflects, in terms of number of exits, the inertia of the activity of the colony (how the number of exits *N*_*t*_ is affected by the number of exits at *t*-1), whereas the variation in the terms γ_1_(*d*_*t*_, *t*) and γ_2_(*d*_*t*_, *t*) provides information about the responses of the colony to changes in environmental factors (computed number of exists every 5 min. in the morning and afternoon, respectively). The model was fitted to each time series of the numbers of exits using the ‘tsglm’ function in the R package tscount. For each colony, the fitting process yield estimates for α, β, for each monitoring day *d*_*t*_, and the morning and afternoon effects, γ_1_(*d*_*t*_.) and γ_2_(*d*_*t*_.), respectively, were evaluated. Here, the trial effect (set of 5 consecutive days under the same thermal condition) is embedded in the day effect.

Variations in the proportion of forager types (pollen, nectar, water and empty) were analysed using a generalized linear mixed model with a binomial distribution. The model with the lowest sample size-corrected Akaike’s Information Criterion was selected. The temperature treatment was analysed as a fixed factor, whereas colony and a nested effect with run, day and trial were analysed as random factors. The term run defines the sampling order within each afternoon (run 1, 2, 3) and the term day corresponds to the day number after the beginning of treatment (SHW+ and SHW−) within a trial (day 2, 3, 4 and 5).

Variation in nectar parameters (volume, concentration and quantity of sugar), physiological parameters (gene expression, sugar and glycogen levels) and brood size were analysed using repeated measures ANOVA followed by Tukey’s post hoc comparison. Treatments and sampling frames were analysed as fixed factors, whereas the replicate and colony origin were analysed as random factors. The link between vitellogenin and DWV was analysed using Pearson’s correlation test.

## Results

### Thermoregulation

**Figure 1:**
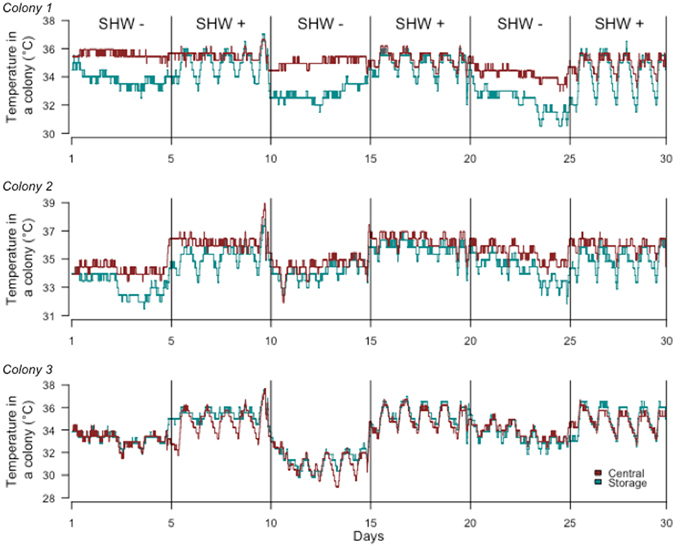
Variations in colony temperature. Temperature recorded on central (red) and storage frames (blue) in each colony. Temperature (°C) was recorded on frames over the successive periods of the SHW treatments by using thermo-tracers. In colony 1, the temperature sensor on the central frame was near the brood. However, in colonies 2 and 3 the brood changed position over time (adult emergence, egg-laying on others frames or away from the temperature sensor), which explains the lack of thermoregulation near the temperature sensors.

The temperatures recorded on storage frames varied greatly (from 30 to 37 °C) and according to the cycle of temperature regimes (Fig. 1), showing that colonies felt the SHW. The temperature recorded on the central frame of colony 1 showed minimal variation (approximately 35 °C). This was due to the presence of the brood near the temperature sensor, and therefore, an effective brood thermoregulation was maintained regardless of the temperature treatment. The temperatures in colony 2 and 3 central frames exhibited greater variation according to the SHW cycles (no brood near the temperature sensor). Brood size, which was estimated to 11,428 ± 2,698 brood cells per colony during the experimental period, remained stable and was not impacted by the exposition to SHW (P = 0.26).

### Impact of heat waves on colony activity

The model for the temporal dynamics of the number of exits was fitted to time series collected from colonies exposed to SHW and from the environmental control colony. We obtained a satisfactory goodness-of-fit (the coefficient of variation R^2^ ranges from 0.95 to 0.97 for the 4 colonies; see also Supplementary Fig. S1, which shows that the observed time series and the predicted time series closely overlap). The basic number of exits α (computed number of exits outside the period [8:00–20:00]) was rather low (from approximately 1 to 6 exits every 5 minutes, Table 1), and even though there was a difference between the control and treated colonies, this difference was negligible compared to the average number of exits observed over the course of the experiment (approximately 160 exits every 5 minutes, Supplementary Fig. S1). The auto-correlation coefficient β (inertia of the colony activity) was consistent over the four series (Table 1), indicating a similar dynamic between colonies. Its high estimated value (approximately 0.93) showed that the inertia of the colony activity was rather high.

**Table 1:** Estimates of basic number of exits (α) and auto-correlation parameter (β) from the autoregressive conditional Poisson model.

Colony	Parameter	Estimate	Std. Error	CI(lower)	CI(upper)
Environmental control	α	5.94	0.14	5.66	6.21
	β	0.924	0.002	0.921	0.928
Colony 1	α	1.44	0.04	1.36	1.52
	β	0.928	0.002	0.924	0.932
Colony 2	α	2.10	0.06	1.98	2.21
	β	0.933	0.002	0.929	0.937
Colony 3	α	1.44	0.04	1.36	1.52
	β	0.936	0.002	0.932	0.940

The α and β parameters were indicated with their standard errors and lower and upper bounds of their confidence intervals at 95% for each colony.

**Figure 2:**
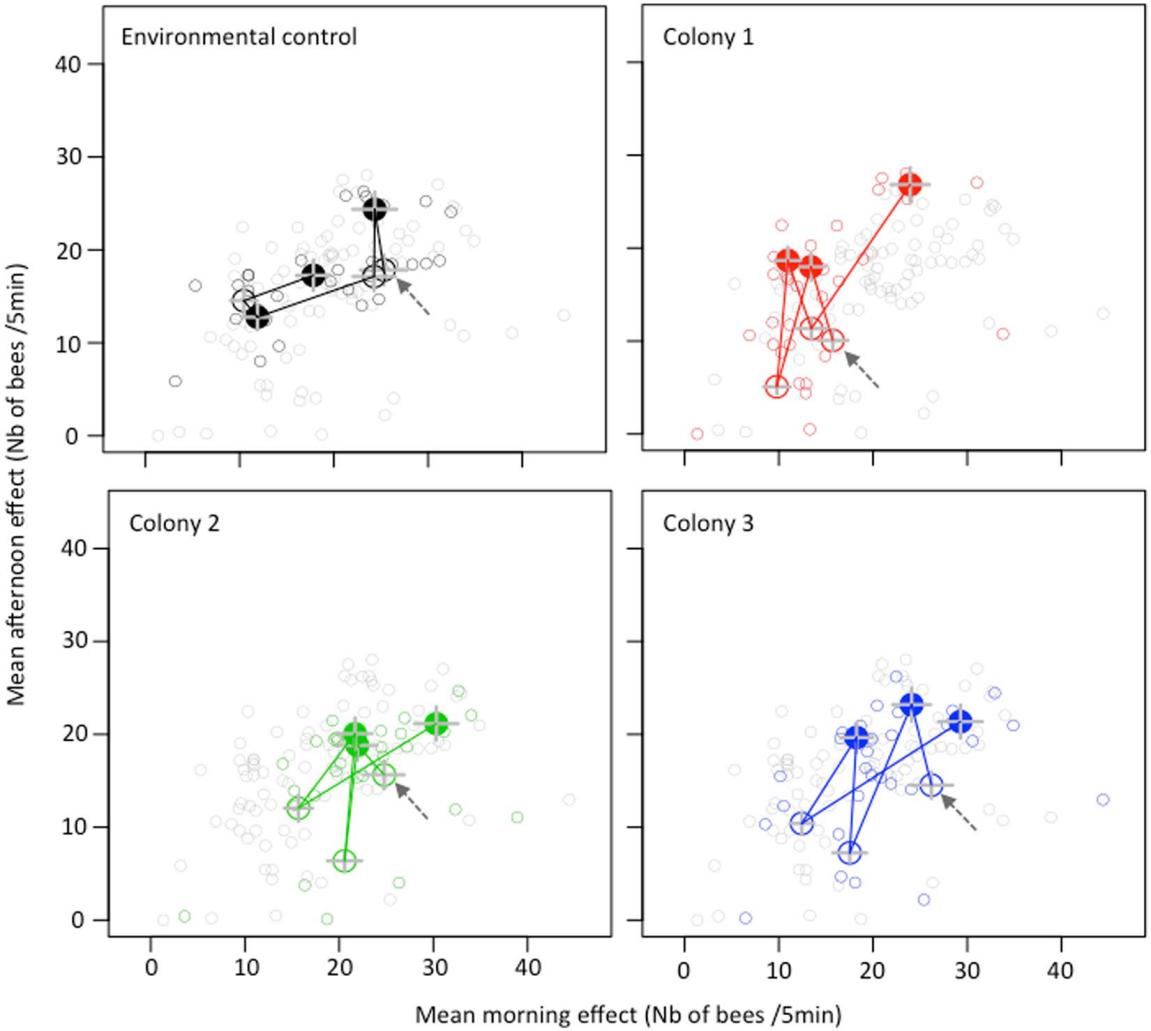
Mean morning effects versus mean afternoon effects estimated from the autoregressive conditional Poisson model. Each small circle gives the morning effect (abscissa) and the afternoon effect (ordinate) estimated for a specific day and colony (computed number of exits every 5 min in the morning and afternoon, respectively). In each panel, small coloured circles (in black, red, green or blue, depending on the panel) correspond to daily morning and afternoon effects of the colony of interest, whereas grey small circles correspond to daily morning and afternoon effects of the three other colonies. In each panel, large circles represent the average effects of the colony of interest for 5-day periods without SHW. Large dots represent the average effects for 5-day periods with SHW. Lines linking the large circles and dots represent jumps from a 5-day period to the following 5-days period. Arrows indicate the first 5-day periods of the experiment without SHW (large circle), which is followed by a 5-day periods with SHW (large dot) and so on. For experimental colonies, large dots (SHW+ periods) were consistently above large circles (SHW− periods), showing an increase in colony activity under SHW in the afternoon.

Regarding the SHW influence on flight activity, we found that morning (γ_1_(*d*_*t*_, *t*)) and afternoon (γ_2_(*d*_*t*_, *t*)) effects ranged from 3 to 45 exits over 5 minutes (mean = 17.7; Fig. 2) and represented 11% of the average number of exits. For colonies exposed to the temperature treatment, the afternoon effects was larger during SHW compared to periods without SHW (large dots above large circles). As opposed to the afternoon, no consistent change according to SHW treatment was observed in the colony activity in the morning. Thus, the temperature treatment induced an increase in the colony activity in the afternoon. In addition, jumps between successive 5-day periods were large and consistent for colonies exposed to the temperature treatment (mean jump = 12.6 ± 0.6) compared to the environmental control colony (mean jump = 7.4 ± 1.6). Thus, the temperature treatment induced larger perturbations in the colony activity.

### Impact of heat waves on foraging behaviour

**Figure 3:**
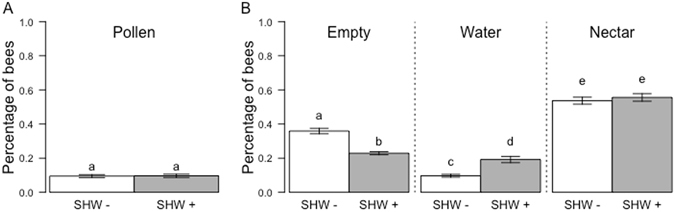
Proportion of pollen, nectar, water foragers and empty bees according to the SHW treatments. The mean percentage and standard error of bees foraging pollen (**A**) and other resources (**B**) are shown for bees sampled during an SHW− (white bars) or SHW+ period (grey bars). For each forager category, different letters indicate significant differences (GLMM).

Regarding the proportion of pollen foragers, the model with the lowest AICc was a null model (model without an explicative variable; null model: 1464.2; model with SHW: 1466.3), indicating that SHW exposure did not impact pollen foraging at the colony level (Fig. 3A).

Regarding the proportion of regurgitating bees, the selected model underlined a significant effect of the treatment with an increase of 15.58% in regurgitating bees during SHW (null model: 1143.2; model with SHW: 1116.5; Fig. 3B). When considering the proportion of water foragers, the model with the lowest AICc underlined an impact of SHW; the proportion of water foragers doubled with SHW exposure (null model: 1013.8; model with SHW: 993.1; Fig. 3B). For nectar foragers, the null model was selected, indicating no impact of SHW on the proportion of nectar foragers (null model: 1284.5; model with SHW: 1286.6). However, SHW induced significant changes in the volume and the concentration of collected nectar. On one hand, the volume of nectar collected by bees slightly increased with SHW treatments (9.47 ± 0.16 and 10.03 ± 0.17 µL before and after SHW, respectively (P < 0.001)), but on the other hand, the saccharose concentration decreased (43.19 ± 0.28 and 38.74 ± 0.25% before and after SHW, respectively (P < 0.001)). Overall, the amount of collected sugar per bee did not change with SHW treatment (4.10 ± 0.07 µg and 3.92 ± 0.07 µg before and after SHW, respectively (P = 0.29)).

### Impact of heat waves on bee physiology

#### Energetic resources

**Figure 4:**
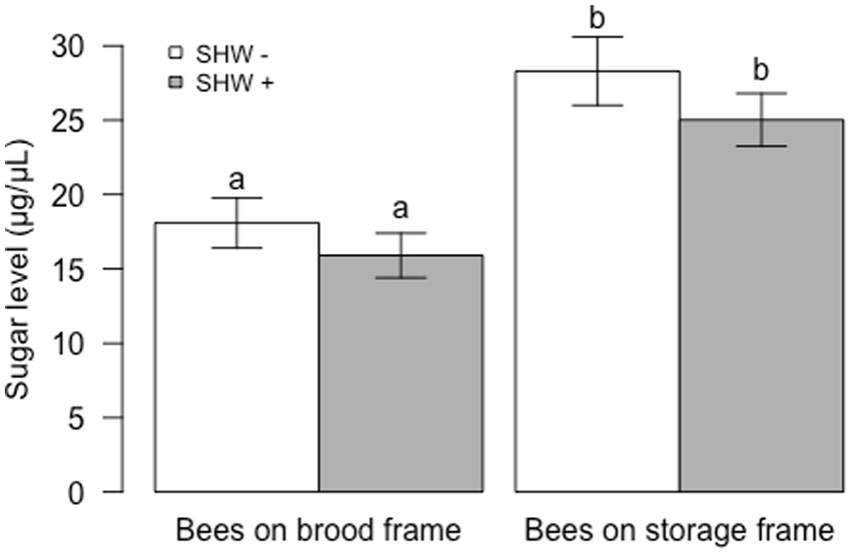
Haemolymph sugar levels according to the SHW treatments. Mean and standard error of sugar levels (µg/µL) in bees sampled at the end of an SHW− (white bars) or SHW+ period (grey bars) (n = 53–54 bees per conditions). The sugar level was higher in bees sampled on storage frames than on brood frames (P < 0.001) but was not affected by SHW (P = 0.17). Different letters indicate significant differences (ANOVA).

A significant difference in total circulating sugar levels was found between bees sampled on brood and storage frames (bees on storage frames had a higher sugar level than bees sampled on brood frames, P < 0.001; Fig. 4). This change was demonstrated by 2x higher levels of glucose and fructose in bees sampled on storage frames, while the trehalose level remained unchanged (Supplementary Fig. S2). However, SHW did not affect total and individual sugar levels in haemolymph (P > 0.08 for total and individual sugar levels).

**Figure 5:**
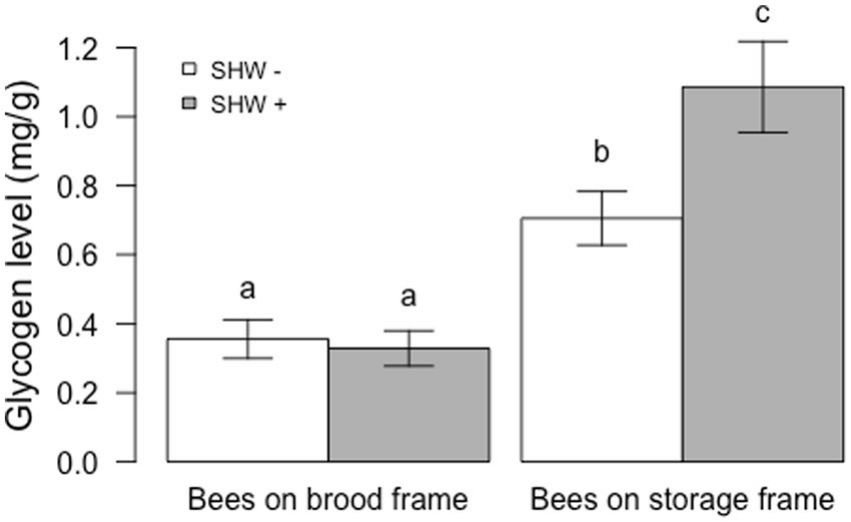
Glycogen levels in whole bees according to the SHW treatments. Mean and standard error of glycogen levels (mg/g) in bees sampled at the end of an SHW− (white bars) or SHW+ period (grey bars) (n = 53–54 bees per conditions). The glycogen level was higher in bees sampled on storage frames than on brood frames (P < 0.001) and increased with SHW for bees sampled on storage frames (P = 0.007). Different letters indicate significant differences (ANOVA followed by Tukey’s post hoc comparisons).

Similarly, glycogen levels were significantly higher in bees sampled on storage frames than in bees sampled on brood frames (P < 0.001, Fig. 5). A significant effect of SHW on glycogen level was also detected (P = 0.032), as well as an interaction between the SHW treatment and the sampled frames (P = 0.014). Though glycogen levels did not change with SHW in bees sampled on brood frames (P = 0.99), it significantly increased in bees sampled on storage frames (P = 0.007).

#### Immune gene expression and DWV loads

**Figure 6:**
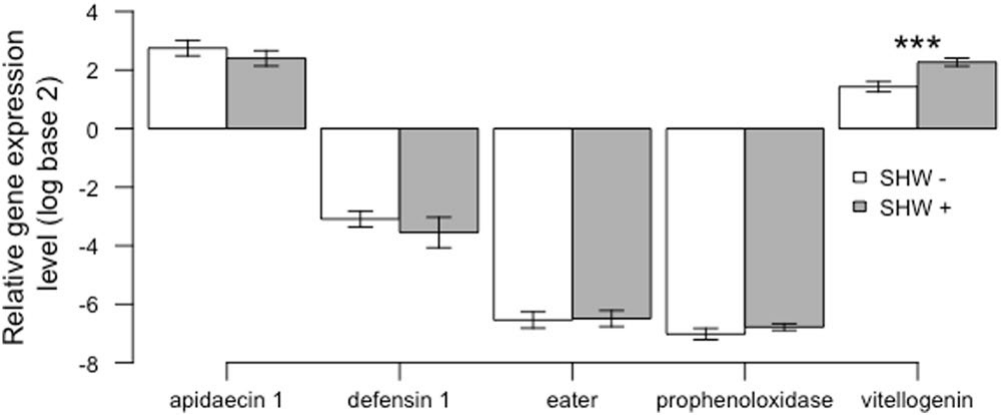
Gene expression levels according to the SHW treatments. Mean and standard error of relative gene expression levels of apidaecin 1, defensin 1, eater, prophenoloxidase and vitellogenin in bees sampled at the end of an SHW− (white bars) or SHW+ period (grey bars) (n = 27 pool of bees per treatment). Significant differences between groups are indicated (***P < 0.001, ANOVA).

**Figure 7:**
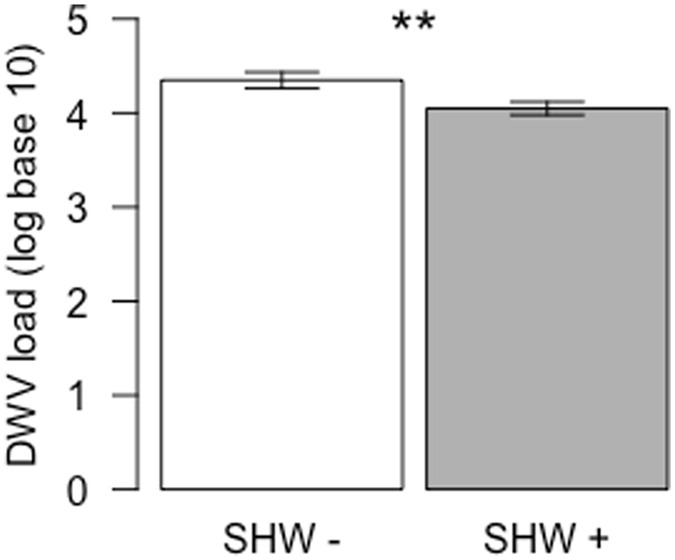
Deformed wing virus loads according to the SHW treatments. Mean and standard error DWV load in bees sampled at the end of an SHW− (white bars) or SHW+ period (grey bars) (n = 26 pool for SHW−; n = 25 pools for SHW+). Significant differences between groups are indicated (**P < 0.01, ANOVA).

The gene expression levels of apidaecin 1, defensin 1, eater and prophenoloxidase were not affected by SHW (P > 0.17 for all genes; Fig. 6). However, vitellogenin expression levels significantly increased with SHW treatment (P < 0.001; Fig. 6 and Supplementary Fig. S3). All colonies were DWV-positive, and we observed a significant decrease in DWV loads after exposing a colony to SHW (P = 0.003; Fig. 7 and Supplementary Fig. S4). Furthermore, a significant negative correlation between DWV loads and vitellogenin levels was detected before the SHW treatments (cor = −0.42, P = 0.034), but this correlation disappeared after the SHW treatments (cor = 0.045; P = 0.83).

## Discussion

Though adult bees can tolerate a large range of temperatures, they need to control the brood rearing temperature with the utmost precision for its survival and normal growth^16, 17^. The mechanism underlying the maintenance of colony thermal homeostasis has been well studied^22, 23^, but whether and to what extent that requires social and physiological costs has been poorly investigated. Answering those questions is essential since climate change is expected to lead to an increase in the severity and frequency of extreme weather events and might extend the list of the numerous pressures to which honeybees are exposed to^46^.

Thanks to optic bee counters and mathematical modelling, for the first time, we could quantify the daily foraging activity at the colony level; we observed a 70% increase in forager traffic in response to a simulated heat wave. The effects were visible in the afternoon when the peak temperature was reached. Since foraging activity was based on the number of exits per bout of 5 minutes, it is reasonable to attribute the traffic increase to a higher number of foragers rather than to a higher number of trips per forager within 5 minutes. Even more, it is less likely that bees performed at least two foraging trips (including the resource distribution around the hive) within 5 minutes. As social insects, when the need of a resource increases, workers are usually recruited from a pool of previously inactive individuals^47–49^. This pool can be large in honeybees since 50% or more of adult bees can be inactive^25, 49^. The bees that do not forage or rarely serve as a backup foraging force were likely to be recruited in response to a SHW.

Next, we focused on the type of resources collected by bees in response to SHW. We found that the proportion of pollen foragers was not impacted by SHW (9.5% and 9.7% for the SHW− and SHW+ periods, respectively), indicating that pollen flow was maintained at the colony level. This is crucial to the colony since a reduction of pollen availability would compromise the physiological development of nurses and brood rearing^50, 51^. However, more than double the number of water foragers (9.7% to 19.2%) were identified in response to SHW, confirming a previous study, which showed an increase in water foragers after exposing the brood nest to heat stress^52^. This intensification of water collection did not occur at the expense of nectar foraging, which remained stable through the experiment but was associated with a decline in the rate of empty bees returning to the hive (from 35.9% to 22.9%). According to Pankiw and Page^53^, empty bees are “nectar foragers that failed to find a source of nectar with a sucrose concentration in excess of their response thresholds”. Since the same authors found that water foragers are the workers with the lowest response threshold to sucrose, followed by pollen foragers, nectar foragers and bees returning to the colony empty (water < pollen < nectar < empty), it is likely that the SHW lowered the sucrose response threshold of bees and therefore increased the proportion of water foragers. This is further supported by the decrease in average sucrose concentration of nectar collected by foragers under SHW, although the range of sugar concentration did not change (from 0 to 75% during SHW+ periods and from 0 to 73% during SHW− periods, see also^52^). In summation, SHW would both stimulate the recruitment of foragers and lower the sucrose response threshold of bees to supply the colony with enough water and enable brood thermoregulation.

Interestingly, though on one hand, the sucrose concentration of nectar collected by bees decreased, on the other hand, its volume was higher under SHW, giving no significant change in the amount of sucrose collected per bee. In contrast, it seems that the SHW treatment increased the nectar and pollen flow of colonies since the forager traffic was almost doubled, and the rate of nectar and pollen forager was not altered. This global increase in resource flow (notably nectar) might be a response to a higher energetic demand required by colony thermoregulation (increase in water collection and brood ventilation).

The next objective was to determine whether SHW modified the level of energetic resources in individual bees. We observed that, before SHW treatment, bees sampled on brood frames had two times less glycogen than bees sampled on storage frames. The fact that nurses stored approximately 50% less glycogen than foragers^54, 55^ suggested that the bees sampled on brood frames had a nurse-like profile, and inversely, bees sampled on storage frames had a forager-like profile. Though the level of glycogen did not change with SHW in bees sampled on brood frames, it increased significantly in bees sampled on storage frames (+54%). Since glycogen is a major source of energy for flight muscles, this change might reflect an adaption to increased activity (foraging flights, brood ventilation) and/or a higher proportion of bees with a forager-like profile on the storage frames. This last assumption is supported by the recruitment of foragers with SHW (see above). Similarly, the level of circulating sugar in haemolymph, partly originating from glycogen catabolism, was significantly higher in bees sampled on storage frames than in bees sampled on brood frames, but it did not change according to the SHW treatment. Ventilation and foraging activity being highly energy-consuming behaviours, this might indicate a quick use of haemolymph sugar by the flight muscles. Overall, the data are in line with the changes observed in the colony foraging activity, but do not reveal any cost of SHW on bee energetic resources.

In insects, the rate of biochemical reactions can be affected by environmental temperatures, and usually, a higher rate of enzymatic activity within physiological limits is observed with an increased temperature^56^. For example, the optimal activity of the phenoloxidase enzyme is reached at temperatures above environmental temperatures^57, 58^, indicating that higher temperatures can enhance its performance. Consistent with this, several studies reported an improvement of immune function at higher temperatures^29, 59^. Even though temperature is controlled on brood frames, there are still some variations on (colony 1: 33 to 36 °C) or near the brood (colonies 2 and 3: 29 to 37 °C) (Fig. 1), and bees can move from one frame to another (storage frames exhibited the highest temperature variability). We therefore expected some changes in immunocompetence, but none of the tested immune genes exhibited changes in their expression level with SHW in bees sampled on brood frames. However, vitellogenin showed a higher expression level, which could reflect an improvement of immune^36^ and antioxidant capacity^60^ or a transition to a more nurse-like profile^36^ to increase brood care. The fact that we observed, at the colony level, a higher proportion of foragers and no decrease in the glycogen levels of bees sampled on brood frames (as it would if bees switched to a more nurse-like profile) tends to exclude the last hypothesis. This could reveal an increased protection against oxidative stress since increased enzymatic rates in insects can lead to higher metabolic rates^56^. In addition, this hypothesis is consistent with our previous results^42^, showing a vitellogenin increase in bees whatever their task specialization (nurse, guard and forager) in response to a heat stress in laboratory conditions.

Moreover, we found that DWV loads decreased with SHW treatment, suggesting potential effects of environmental temperatures on viral population in honeybees. Temperature variation has been shown to influence the resistance of hosts to viruses in insects^30, 31^, but this DWV decline was not associated with enhanced immune functions. Indeed, the expression of immune genes did not increase, and though DWV loads were correlated to vitellogenin levels before SHW, this correlation disappeared with the SHW treatment. Our data suggest the existence of alternative physiological functions inhibiting virus multiplication or a direct thermal inhibition. In insects, a temperature dependence of host susceptibility to viral infections has been demonstrated several times. For example, in silkworm, thermal inhibition of viral diseases has been attributed to a limited accumulation of infectious progeny and the replication mechanism of the virus^31^. In crickets, the protective effects of high temperature are caused by the induction of heat-shock proteins in uninfected cells^61^. Similar mechanisms may be speculated in honeybees.

A limit of our study was that heat waves were simulated only at the colony level and not at the field level. Though high field temperature might on one hand improve forager flight performance^62^ owing to lower flight metabolic rate^63^, on the other hand, it may lower the availability of water and flower nectar^64^. Therefore, future studies should take into account this trade-off. Nevertheless, we showed that honeybee colonies could remarkably adapt to heat waves but most importantly without being at the expense of the colony nectar and pollen flow. No cost was detected at the individual level, and SHW was rather beneficial regarding viral infection, although the impact of climate change on bee diseases should be studied long-term^65^. The solicitation of the backup foraging or other task forces might however be costly if the colony is subjected to other environmental pressures. For example, a decrease in the availability or size of those backup forces might limit the colony capacity to buffer great field mortality. This scenario could certainly be generalized to any environmental stressors that trigger the recruitment of bees for a specific task (e.g. foragers), and not only in response to temperature increase. Finally, quantifying honeybee colony responses to climatic variations will help to model and further predict their dynamic and/or survival under different climate change scenarios (Switanek *et al*.)^66^.

## Electronic supplementary material

Supplementary Information
