# Supplementary material for: Colony adaptive response to simulated heat waves and consequences at the individual level in honeybees (Apis mellifera)
Source: Sci Rep. 2017 Jun 19;7:3760. doi: 10.1038/s41598-017-03944-x (PMC5476575; doi:10.1038/s41598-017-03944-x)

Colony adaptive response to simulated heat waves and consequences at the individual level in honeybees (*Apis mellifera*)

Célia Bordier, Hélène Dechatre, Séverine Suchail, Mathilde Peruzzi, Samuel Soubeyrand, Maryline Pioz, Michel Pélissier, Didier Crauser, Yves Le Conte and Cédric Alaux

Table S1: Primer pairs used for quantitative PCR of honeybee genes and virus.

Locus	Identifier	Forward primer	Reverse primer	Reference
Actin	GB17681	TGC CAA CAC TGT CCT TTC TG	AGA ATT GAC CCA CCA ATC CA	Amdam et al., 2004[1]
Apidaecin 1	GB47546	TTT TGC CTT AGC AAT TCT TGT TG	GTA GGT CGA GTA GGC GGA TCT	Gregorc et al., 2012[2]
Defensin 1	GB19392	TGC GCT GCT AAC TGT CTC AG	AAT GGC ACT TAA CCG AAA CG	Gregorc et al., 2012[2]
Eater	GB50508	CAT TTG CCA ACC TGT TTG T	ATC CAT TGG TGC AAT TTG G	Simone et al., 2009[3]
eIF3-S8	GB12747	TGA GTG TCT GCT ATG GAT TGC AA	TCG CGG CTC GTG GTA AA	Ament et al., 2011[4]
PPO	GB43738	AGA TGG CAT GCA TTT GTT GA	CCA CGC TCG TCT TCT TTA GG	Evans, 2006[5]
Vitellogenin	GB13999	TTG ACC AAG ACA AGC GGA ACT	AAG GTT CGA ATT AAC GAT GAA	Fischer and Grozinger, 2008[6]
DWV	AY224602	TTC ATT AAA GCC ACC TGG AAC ATC	TTT CCT CAT TAA CTG TGT CGT TGA	Locke et al., 2012[7]

1. Amdam, G. V., Norberg, K., Fondrk, M. K. & Page, R. E. Reproductive ground plan may mediate colony-level selection effects on individual foraging behavior in honey bees. *Proc. Natl. Acad. Sci. U. S. A.* **101,** 11350–5 (2004).
2. Gregorc, A., Evans, J. D., Scharf, M. & Ellis, J. D. Gene expression in honey bee (*Apis mellifera*) larvae exposed to pesticides and Varroa mites (*Varroa destructor*). *J. Insect Physiol.* **58,** 1042–1049 (2012).
3. Simone, M., Evans, J. D. & Spivak, M. Resin collection and social immunity in honey bees. *Evolution (N. Y).* **63,** 3016–3022 (2009).
4. Ament, S. A. *et al.* Mechanisms of stable lipid loss in a social insect. *J. Exp. Biol.* **214,** 3808–3821 (2011).
5. Evans, J. D. Beepath: An ordered quantitative-PCR array for exploring honey bee immunity and disease. *J. Invertebr. Pathol.* **93,** 135–139 (2006).
6. Fischer, P. & Grozinger, C. M. Pheromonal regulation of starvation resistance in honey bee workers (*Apis mellifera*). *Naturwissenschaften* **95,** 723–729 (2008).
7. Locke, B., Forsgren, E., Fries, I. & de Miranda, J. R. Acaricide treatment affects viral dynamics in *Varroa destructor*-infested honey bee colonies via both host physiology and mite control. *Appl. Environ. Microbiol.* **78,** 227–235 (2012).

Figure S1: Fit of the autoregressive conditional Poisson model to the time series. Data are shown for the environmental control colony and for the colonies exposed to SHW (Colonies 1, 2 and 3). Black curve is the observed number of exits. Red curve is the predicted number of exits. Green curve represents the morning effects. Blue curve represents the afternoon effects. Hatched periods are 5-day periods with simulated heat waves for colonies exposed to the temperature treatment.

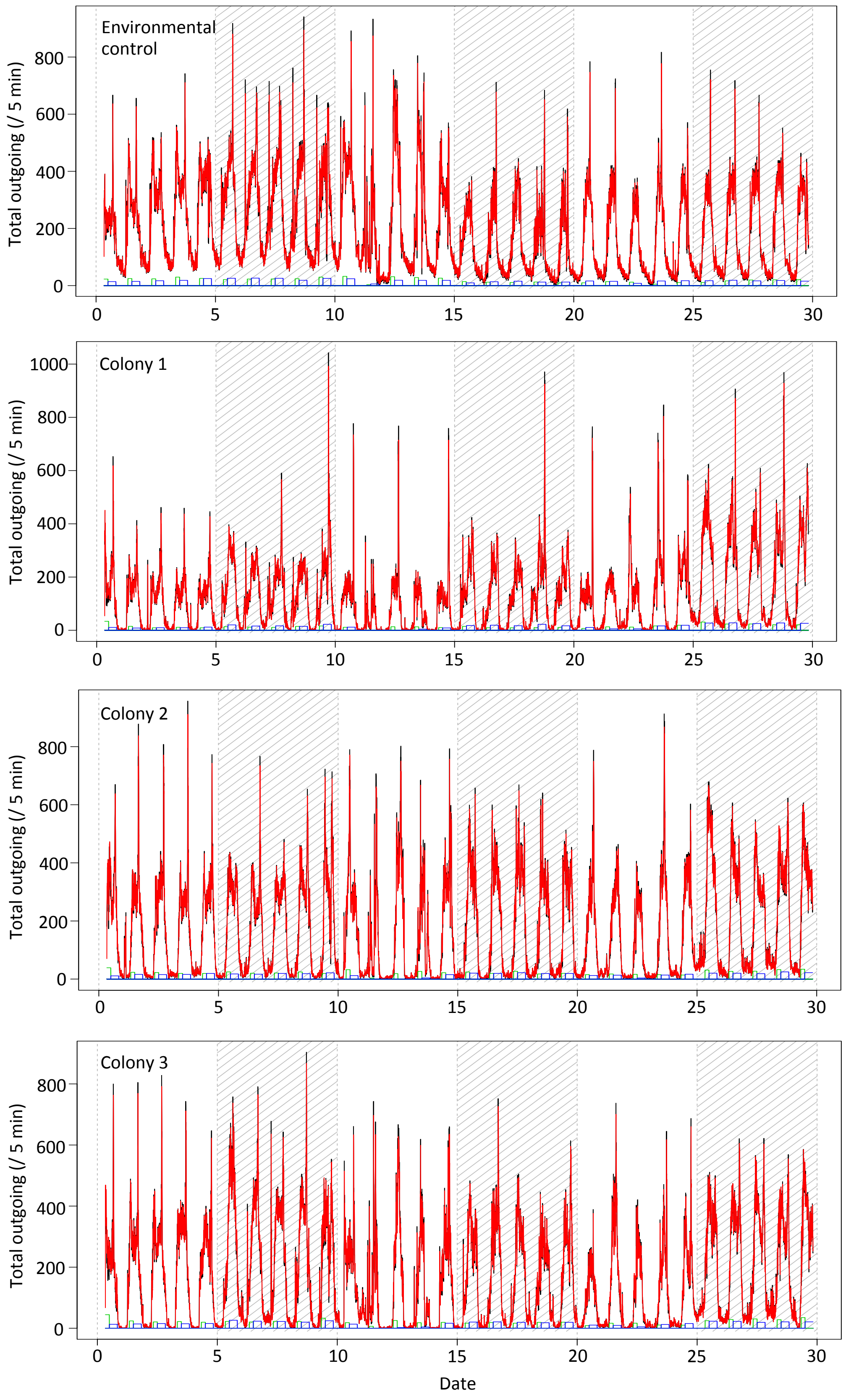

Figure S2: Fructose, glucose and trehalose levels in bee haemolymph. Mean and standard error of fructose, glucose and trehalose levels (µg/µL) in bees sampled on brood frames or storage frames at the end of an SHW- (white bars) or SHW+ period (grey bars) (n = 53-54 bees per conditions). For each sugar, different letters indicate significant differences between each group (ANOVA).

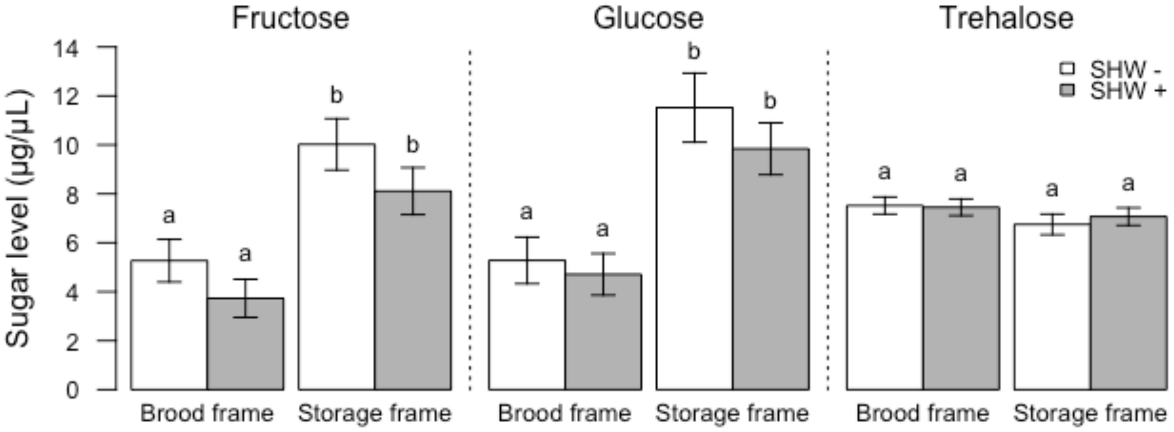

Figure S3: Vitellogenin expression levels over the successive SHW periods. Data are shown according to the SHW treatment in bees sampled at the end of an SHW- (white boxes) or SHW+ period (grey boxes) (n = 9 pool of bees per group). Boxes show the 1st and 3rd interquartile range, and the line denotes the median. Whiskers encompass 90 % of the individuals; each outlier is represented by a circle. Vitellogenin expression levels significantly increased with the SHW treatment (ANOVA: $P < 0.001$).

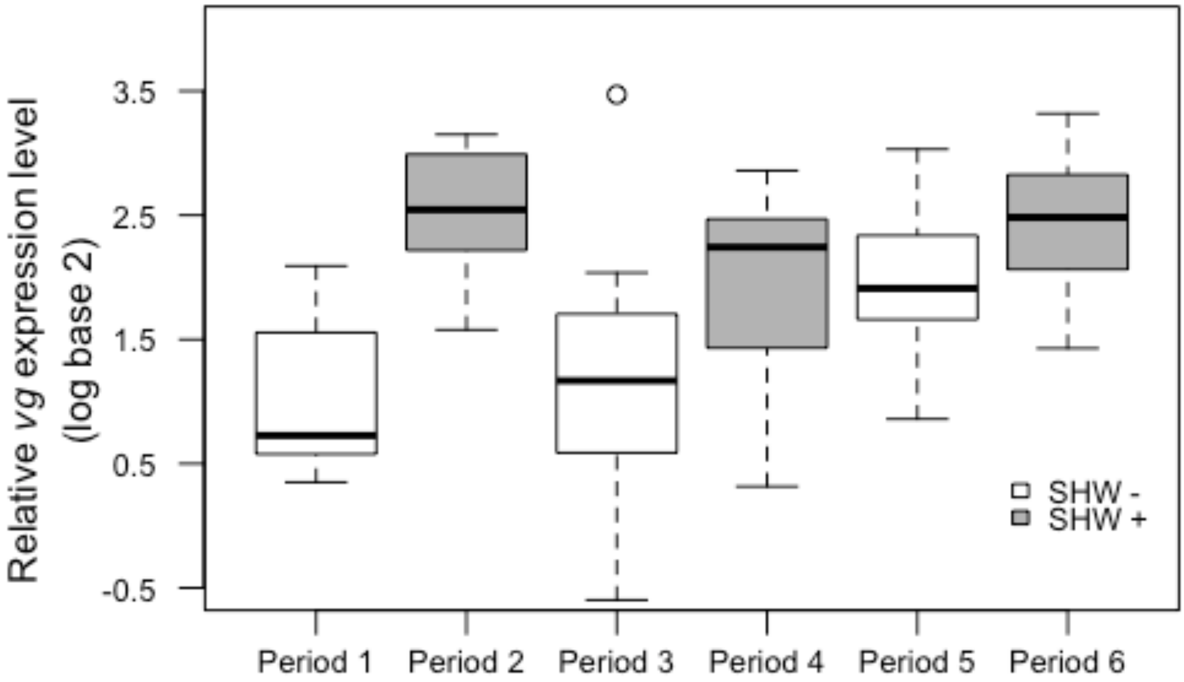

Figure S4: Deformed wing virus load over the successive SHW periods. Data are shown according to the SHW treatment in bees sampled at the end of an SHW- (white boxes) or SHW+ period (grey boxes) (n = 8 - 9 pool of bees per group). Boxes show the 1st and 3rd interquartile range, and the line denotes the median. Whiskers encompass 90 % of the individuals; each outlier is represented by a circle. DWV loads significantly decreased with SHW treatment (ANOVA: P = 0.003).

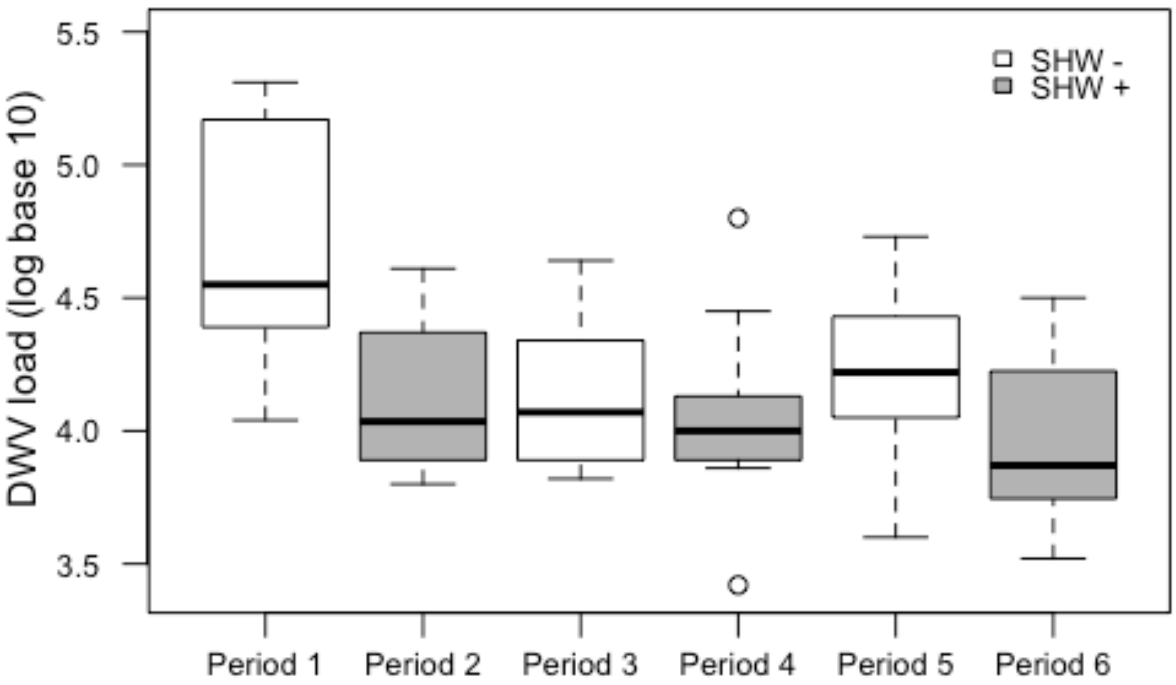

Supplement: Supplementary file 1 — Supplementary Information [file 41598_2017_3944_MOESM1_ESM.pdf]
